# Cost effectiveness analysis of implementing tuberculosis screening among applicants for non-immigrant U.S. work visas

**DOI:** 10.1186/s41479-020-00078-z

**Published:** 2020-12-25

**Authors:** Bisma Ali Sayed, Drew L. Posey, Brian Maskery, La’Marcus T. Wingate, Martin S. Cetron

**Affiliations:** 1 Division of Global Migration and Quarantine, Centers for Disease Control and Prevention, 1600 Clifton Road, Building 16, MS 16-4, Atlanta, GA 30329 USA; 2 College of Pharmacy, Howard University, Washington, DC USA

**Keywords:** Tuberculosis, Screening, Migration, Cost effectiveness

## Abstract

**Background:**

While persons who receive immigrant and refugee visas are screened for active tuberculosis before admission into the United States, nonimmigrant visa applicants (NIVs) are not routinely screened and may enter the United States with infectious tuberculosis.

**Objectives:**

We evaluated the costs and benefits of expanding pre-departure tuberculosis screening requirements to a subset of NIVs who arrive from a moderate (Mexico) or high (India) incidence tuberculosis country with temporary work visas.

**Methods:**

We developed a decision tree model to evaluate the program costs and estimate the numbers of active tuberculosis cases that may be diagnosed in the United States in two scenarios: 1) “Screening”: screening and treatment for tuberculosis among NIVs in their home country with recommended U.S. follow-up for NIVs at elevated risk of active tuberculosis; and, 2) “No Screening” in their home country so that cases would be diagnosed passively and treatment occurs after entry into the United States. Costs were assessed from multiple perspectives, including multinational and U.S.-only perspectives.

**Results:**

Under “Screening” versus “No Screening”, an estimated 179 active tuberculosis cases and 119 hospitalizations would be averted in the United States annually via predeparture treatment. From the U.S.-only perspective, this program would result in annual net cost savings of about $3.75 million. However, rom the multinational perspective, the screening program would cost $151,388 per U.S. case averted for Indian NIVs and $221,088 per U.S. case averted for Mexican NIVs.

**Conclusion:**

From the U.S.-only perspective, the screening program would result in substantial cost savings in the form of reduced treatment and hospitalization costs. NIVs would incur increased pre-departure screening and treatment costs.

**Supplementary Information:**

The online version contains supplementary material available at 10.1186/s41479-020-00078-z.

## Background

Almost a third of the world’s population is estimated to be infected with *Mycobacterium tuberculosis*, the bacterial cause of tuberculosis (TB) [1]. In the United States, TB incidence among the non-U.S.-born population is 13 times higher than among the U.S.-born population. Immigration from countries with high TB incidence maintains the close link between TB burden and disease incidence inside and outside the United States and supports the need to strengthen TB control efforts abroad [2].

The Public Health Service Act authorizes the Centers for Disease Control and Prevention (CDC) to prevent the introduction, transmission, and spread of specific communicable diseases in the United States, including infectious TB [3]. CDC’s Division of Global Migration and Quarantine (DGMQ) has developed requirements for TB screening, which are outlined in the TB Screening and Treatment Technical Instructions (TB TIs) [4]. Applicants for immigrant or refugee visas that lead to permanent residence in the United States, must undergo medical examinations (42 CFR 34) by panel physicians in accordance with the TB TIs [4]. Panel physicians are licensed physicians in the country of departure who have an agreement with the U.S. consular section to perform the examination.

Immigrant medical examinations include a medical history, physical examination, and chest radiograph (CXR) for applicants ≥15 years of age. If the applicant has a CXR suggestive of TB, signs and symptoms of TB, or known HIV infection, sputum specimens are tested: smears for acid-fast bacilli and culture [4]. Immigrant-visa applicants with active TB are required to complete treatment before they are admissible to the United States [4]. Immigrant-visa applicants who have negative cultures are given a Class B1 TB classification and advised to follow up with local public health departments (PHDs) in the United States [4]. The results of their pre-departure medical examinations are transmitted via the Electronic Disease Notification System (EDN) to the PHDs in the U.S. jurisdictions where they will live [5]. See Appendix Section A1.

Unlike applicants for permanent residence, applicants for nonimmigrant visas (NIVs) are not routinely required to undergo TB screening. Annually, approximately 9 million NIVs are issued nonimmigrant visas that authorize temporary U.S. residence for business, education, or other purposes [6].

This volume of NIVs is substantially greater than the 500,000 to 700,000 immigrants and refugees who must undergo TB screening before they are issued U.S. immigrant visas [6–8]. During 2001–2008, a modeling analysis estimated that 11,500 non-U.S.-born individuals living in the United States were infected with active TB during their first year after arrival. The majority of cases (6717) estimated to occur in the first year after arrival were among NIVs including an estimated 4211 cases in temporary workers and student/exchange visitors who would be expected to stay for longer than 6 months [9]. This modeling analysis does not account for the undocumented non-U.S.-born population. Undocumented individuals were found to comprise 19.2% of tuberculosis cases occurring among a sample of non-U..S.-born tuberculosis patients diagnosed in the United States within 6 months of their arrival and 21.4% of cases diagnosed among non-U.S.-born patients diagnosed more than 6 months after U.S. arrival [10, 11].

**Fig. 1:**
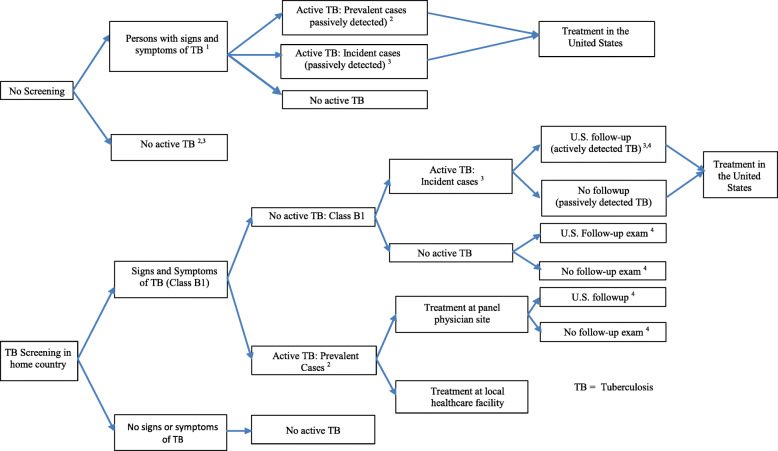
Decision Tree Model Comparing a Screening Program for Nonimmigrant Visa Applicants to No Screening Notes: Decision model does not display all nodes. Each cohort proceeds through the screening and no-screening arms of the model. PHD: public health department, TB: tuberculosis, NIV- non-immigrant visa. For the no-screening arm, all cases are diagnosed passively and treated in the United States. All costs accrue in the United States. For the screening arm, costs accrue in applicants’ home countries and in the United States. Costs are incurred for the panel physician medical exams and diagnosis and treatment as necessary. ^1^The probability that an applicant has signs and symptoms of TB is based on the country-specific immigrant rate multiplied by a country-specific correction factor for applying immigrant data to our hypothetical populations. ^2^The number of active TB cases among NIV candidates from India and Mexico is the cohort size multiplied by the fraction with clinical findings suggestive of TB and by the fraction diagnosed with active TB given signs and symptoms. This group of applicants with active TB will not be able to enter the United States until they complete treatment. Among these, the probability that they are treated by panel physicians is applied to determine cost of treatment to the patient via panel physician (opportunity and treatment costs) and to the national government (treatment costs for those treated outside the panel physician). Recruitment and visa costs are incurred by U.S.-based H-1B employers when applicants are treated outside the panel physician site. ^3^The total number of active TB cases in each cohort is the sum of the prevalent cases and incident cases. ^4^Among those with clinical signs or symptoms suggestive of TB, 78.6% of Mexicans and Indians follow up with PHDs after arrival in the United States. Among Class B1 arrivals who did not have active TB, 2.6% of Indians and 0.6% of Mexicans develop incident cases of TB and incur treatment costs in the United States. Such cases will be detected actively if they follow up with a PHD or passively if they do not

To reduce the TB burden in the non-U.S.-born population living in the United States, the federal government’s National Action Plan for Combatting Antibiotic-Resistant Bacteria includes a recommendation to expand the overseas screening program to include nonimmigrants not currently screened from high-TB-burden areas [12]. This economic analysis of TB screening for NIVs before admission to the United States examines the costs and number of active TB cases expected to occur in the United States for two strategies: 1) “Screening” a subset of NIVs seeking long-term work U.S. visas for active TB and treatment of those diagnosed with active TB in their home countries with recommended U.S. follow-up for NIVs at elevated risk of active TB; and, 2) the current baseline policy, “No Screening”, under which this NIV population is not required to undergo screening (see Fig. 1). Under the current baseline, costs accrue only in the United States when active TB patients are diagnosed and treated after arrival. Under the screening strategy, screening and treatment costs would accrue both in NIVs’ home countries and in the United States. Costs were calculated from the multinational perspective (i.e., costs incurred in the home countries and the United States), the U.S.-only perspective (i.e., only costs incurred in the United States and excluding costs incurred in NIVs’ home countries), and subdivided by stakeholder. This analysis is the first to examine the NIV long-term worker population, although previous work has examined U.S.-bound students [13] and other migrants [14, 15].

TreeAge Pro Software (Williamstown, MA) and Microsoft Excel were used to construct the decision tree models and estimate costs in 2013 dollars [16, 17].

## Methods

### Target population and time frame

The screening and treatment costs and number of U.S. TB cases detected for a one-year period were estimated for three populations of NIVs seeking work visas: H-1B visa candidates from India, H-2A visa candidates from Mexico, and H-2B visa candidates from Mexico. H-1B visa candidates are skilled workers often employed in computer-related occupations [18]. H-2A visa candidates are agricultural workers, and H-2B visa candidates are seasonal workers employed in a variety of industries (e.g., fishing, hospitality) [19]. Analyses also include adult family members with H-4 visas who accompany H-1B visa candidates. Children of H-1B visa candidates were excluded for simplicity because the predeparture screening process is different for children and children compose < 3% of the new H-4 visa applicants from India. Children of H-2A and H-2B visa applicants were not eligible for visas to travel with their parents. We restricted our analyses to India and Mexico because these countries have moderate to high rates of active TB and provide the greatest number of temporary workers in the respective visa categories (see Appendix Section A2) [6, 20].

Using the average number of annual visa issuances from 2010 through 2012 (H-1B + H-4 visas for India, H-2A and H-2B visas for Mexico), we constructed hypothetical cohorts for each country (Table 1) [6]. An average of 70,577 H-1B visas and 43,766 adult H-4 visas were issued in India; 55,189 H-2A visas and 35,298 H-2B visas were issued in Mexico [6, 21]. We reduced each country-specific cohort by 10% to adjust for persons who would decide not to enter the United States after receiving visas. The exact number of persons arriving from each country with these visas is unknown. The numbers of issuances should exceed the numbers of arrivals because some applicants may decide to remain in their home country or if hiring priorities for U.S.-based firms shift [31]. See Appendix Section A3. The analysis uses a 1-year time frame under the assumption that most individuals who would be diagnosed with active tuberculosis at pre-arrival screening would develop symptoms within a year of arrival.

**Table 1:** Inputs to a model estimating costs of tuberculosis screening among Indian and Mexican nonimmigrant visa applicants, base case

	India		Mexico		Source
	H-1B (High skilled work visas)	H-4 (Family members of H-1B)	H-2A (Agricultural work visa)	H-2B (Seasonal work visa)	
Average annual visa issuances	70,577	43,766	55,189	35,298	[6, 21]
Hypothetical cohort^a^	63,520	39,389	49,670	31,769	Assumption
Total active TB cases in cohort	138	86	32	20	Calculation
**India and mexico inputs**					
% with clinical findings suggestive of TB^b^	3.35	3.35	2.2	2.2	TB Indicator data
Persons identified as Class B1^c^	2128	1320	1068	683	TB Indicator data
% with active TB among those with clinical findings suggestive of TB	4.0	4.0	2.3	2.3	TB Indicator data
Total number of active TB cases in home countries (prevalent cases)^d^	85	53	25	16	Calculation
% of patients with active TB who enroll at panel physician sites	61	61	77	77	TB Indicator data
Time for screening^e^	1 day	1 day	1 day	1 day	
Time for diagnosis^e^	3 days	3 days	3 days	3 days	
Time spent on treatment at panel physician site^e^	30% of 6 months treatment	30% of 6 months treatment	30% of 6 months treatment	30% of 6 months treatment	
Time spent at a local treatment site^e^	237.5 h	237.5	237.5	237.5	Calculation
**Domestic Inputs**					
% of Class B1s who will develop active TB in the U.S.	2.6	2.6	0.6	0.6	EDN 2011–2014
Number of Class B1s who will develop active TB in the U.S. (incident cases)	53	33	6	4	EDN 2011–2014
% receiving DOT only	62	62	62	62	[22]
% receiving DOT and SAT	29.3	29.3	35	29.3	[22]
% receiving SAT only	8.7	8.7	3.2	8.7	[22]
% receiving DOT, healthcare worker visits patient	60	60	1	60	Assumption
% receiving DOT, patient visits health department	40	40	0	40	Assumption
% hospitalized (active detection)^f^	8	8	8	8	[23]
% hospitalized (passive detection)^g^	49	49	49	49	[23]
% presenting for follow-up at PHDs	78.6	78.6	78.6	78.6	[24]
% receiving a simple follow-up	10.76	10.76	8.46	8.46	EDN 2011–2014
% receiving only chest radiograph	28.44	28.44	39.04	39.04	EDN 2011–2014
% Receiving chest radiograph, smears, and cultures	39.4	39.4	31.1	31.1	EDN 2011–2014
Time for diagnostic visits^e^	2 h	2 h	2.5 h	2 h	
Time for treatment administration^e^					
Healthcare worker visits patient	8 min/visit	8 min/ visit	8 min/visit	8 min/visit	Assumption based on previous study [13]
Patient visits PHD	1 h/visit	1 h/visit	Does not travel	1 h/visit	Assumption based on previous study [13]
Time for follow-up visits^e^	1.5 h/visit	1.5 h/visit	2 h/visit	1.5 h/visit	Assumption based on previous study [13]
Disease impairment	12.5 days	12.5 days	12.5 days	12.5 days	[25]
Hospitalization	14 days	14 days	14 days	14 days	[26]

Notes: Numbers may not add up due to rounding

*TB* Tuberculosis, *DOT* Directly observed therapy, *SAT* Self-administered therapy, *EDN* Electronic Disease Notification System, *NIV* Non-immigrant visa applicant

^a^Cohort size was calculated by averaging the number of country-specific visa issuances over a 3-year period (2012–2013), and reducing by 10% [6]

^b^For India, this parameter is varied in a sensitivity analysis to account potential differences in the Indian immigrant versus NIV rates for signs and symptoms of TB

^c^Number of Class B1 NIVs was computed by subtracting the total number of active TB cases from the total number of with clinical findings suggestive of TB cases

^d^TB cases among persons with Class B1 designations = Total persons with Class B1 designation x proportion of those with B1 designation that are diagnosed at U.S. follow-up

^e^Opportunity costs were estimated for applicants for screening, diagnosis, and treatment using average annual wages for each visa category. Wages were obtained from the Bureau of Labor Statistics (BLS) or international sources. International wages were converted to dollars using PPP. For Indian H-1B workers, 1 h = $37.43 [27]. H-2A and H-2B workers’ wages in Mexico were obtained from International Labour Office (H-2A: 1 h = $2.46. H-2B: 1 h = $3.27) [28]. These wages were not adjusted for benefits. Time costs for Indian family members were based on GDP adjusted for PPP [29]

Total compensation was estimated to be $56.25 per hour [30]. Agricultural workers (H-2A) wages were obtained from the BLS. Total compensation was estimated to be $13.34 per hour [30]. Seasonal workers’ (H-2B) time was valued using average wages for the top five occupations for H-2B foreign certifications [19, 30]. H-2B visa applicants’ wages were reduced by 20% because these workers are paid less than their U.S. counterparts. Total compensation for H-2B visa holders was estimated to be $13.52/h. Total compensation includes benefits

^f^Cases diagnosed actively via public health department follow-up is calculated by multiplying the total number of active cases expected in the population x the percentage that follow up at public health departments

^g^Cases diagnosed passively are for individuals who do not report to public health departments for follow-up and develop active TB

### Estimating the total number of active TB cases expected

In the model, we assumed that all NIVs would be screened prior to arrival in the United States and that NIVs with positive sputum smears or cultures would need to complete treatment prior to receiving visas. In addition, NIVs with clinical findings suggestive of TB, but negative smear/culture results in India and Mexico, would be recommended to follow up with health departments in the United States. The estimated numbers of active TB cases to occur in these populations were calculated using two data sources. TB exam outcomes for immigrant and refugee applicants are submitted to CDC by panel physicians and are known as the Division of Global Migration and Quarantine TB Indicators (DGMQ TB Indicators) [4]. Country-specific DGMQ TB Indicators for immigrants were used to estimate the prevalence of TB in NIV populations. We extracted these data from the DGMQ TB Indicators for 2012–2013.

In addition to cases diagnosed before U.S. entry, incident TB cases in immigrants who arrive with Class B1 status may be diagnosed during follow-up examinations at U.S. PHDs. CDC recommends all immigrants and refugees who arrive with B1 status should follow up with health departments after arrival and follow-up exam outcomes are maintained in a second data set compiled in CDC’s Electronic Disease Notification System (EDN) [5, 24]. We extracted these data from EDN for the period from 2011 to 14.

The third data source consulted for this analysis was CDC’s TB surveillance data collected by the Division of TB Elimination (DTBE) from 2012 through 2013 [22, 32]. The DTBE surveillance data includes all verified U.S. TB cases. For most cases, DTBE also records country of birth and number of years spent in the United States if the person is non-U.S.-born [32]. This data source was used as a check on the application of results from immigrant screening to the NIV population.

First, we applied the percentages of persons examined who were diagnosed with active TB from immigrant-applicant exams (DGMQ TB Indicator data) to our hypothetical NIV cohorts to estimate the number of NIVs who would be diagnosed with active TB in each country and the number that would arrive in the United States with Class B1 status and recommendations for follow-up at PHDs.

After estimating the number from each NIV cohort that would travel to the United States with B1 status, the country-specific rate of active TB among class B1 immigrants undergoing follow-up examination at U.S. PHDs was applied to each NIV cohort to estimate the number of incident cases of active TB. The country-specific rates of incident TB at U.S. follow-up among new arrivals were assumed to be the same for NIVs as for immigrants because only persons with signs and symptoms of TB during predeparture exams (Class B1) were assumed to undergo U.S. follow-up. We assumed that zero incident cases of active TB would be diagnosed among new arrivals without B1 status.

The numbers of cases estimated to occur from the application of immigrant data to the hypothetical cohorts in this model were then compared to DTBE’s U.S. surveillance data. During 2012–2013, there were 1027 cases reported among individuals born in India and 2552 among individuals born in Mexico. These totals include 189 cases (94.5 per year on average) diagnosed in Indians and 220 (110 per year on average) in Mexicans who had been in the United States for less than 1 year [32]. Of note for Mexico, a previous study reported that 25% of tuberculosis patients from Mexico and Latin America self-reported undocumented status [10]. A potential correction factor was applied to the fraction of NIVs presenting at the predeparture exam with signs or symptoms of TB (including abnormal CXR) to account for potential discrepancies between the numbers of cases estimated from applying screening outcomes for immigrants to NIVs. See Appendix Section A3.

### TB control activities and associated costs in the United States

#### Active TB treatment costs

Typically, persons with active TB undergo three visits for the initial diagnosis and five follow-up visits during treatment (Table 2) [15]. Since treatment of active TB at PHDs is generally provided free of charge to the patient, we estimated costs by valuing physician and nurse time using mean annual salaries adjusted for nonwage benefits [30]. Costs for all medical tests were estimated using the 2013 Medicare Clinical Fee Schedule [33]. We added 30% of staff compensation for administrative overhead. Patients’ travel costs were estimated based on fuel prices and average mileage to healthcare facilities as reported in the National Household Travel Survey [34–36]. To estimate patient opportunity time costs, we assumed that each patient spent 2 h for each diagnostic visit and 1.5 h for each follow-up visit. For agricultural workers, we added an extra 30 min for longer commutes [37]. See Appendix Section A4. These time estimates were multiplied by expected wage rates for H-1B, H-2A, and H-2B visa applicants or by average GDP per capita-hour for H-4 visa candidates.

**Table 2:** Costs of tuberculosis medical exam and treatment for visa applicants, by country

Medical components	United States	Cost in USD (2013)	Medical components	India	Mexico
	diagnostic & follow-up visits (quantity)^**a**^			Cost in USD (2013)^**b**^	Cost in USD (2013)^**b**^
Chest radiograph	3	$35.82	Panel physician screening exam	$26.46	$190
Sputum smears	5	$9.99	Chest radiograph	Included in exam fee, $6.00 for subsequent	Included in exam fee
Cultures	5	$20.07	Sputum smears	$32.68	Included in exam fee
Complete blood count with differential	3	$7.93	Cultures	$32.68	Included in exam fee
Serum chemistry	1	$15.71	Treatment at panel physician site	$79.40	Included in exam fee
Drug susceptibility tests	4	$10.50	Additional medical exams^c^	$259.00	Included in exam fee
Hepatic function panel	3	$15.17	Treatment at local healthcare site	60.00	Included in exam fee

*Notes*: In the United States, each person with active tuberculosis receives three diagnostic visits and five follow-up visits. The costs for medical components were computed using the 2013 Physician Fee Schedule from the Centers for Medicare and Medicaid Services

*Diff* differential

^a^Persons with Class B1 TB designation do not receive all diagnostic and follow-up visits unless they are diagnosed with active tuberculosis

^b^All prices obtained from Indian and Mexican panel physicians and are presented in 2013 dollars. We estimated that 30% of the panel physician screening exam costs in India is attributable to TB, and 50% of the panel physician screening exam in Mexico costs is attributable to TB. Note that the higher fraction is assumed for Mexico, because, in India, culture/smear testing and treatment are charged separately only for applicants that need such services. Treatment at panel physician consists of the medication and directly observed therapy

^c^Additional medical exams means the total costs for additional smears, cultures, chest radiographs, drug susceptibility testing, and liver testing at panel physician sites

Medication costs were estimated for a 6-month treatment regimen for an adult weighing 150 pounds. We assumed these cases were responsive to first-line anti-TB drugs [38, 39]. Treatment regimens (directly observed therapy [DOT], self-administered therapy [SAT], and combination therapy [DOT + SAT]) were assumed to vary based on occupation, and costs were adjusted accordingly. See Appendix Section A4.

Travel costs associated with medication administration were estimated for patients and PHDs using national reimbursement rates, average mileage to health departments, and average gasoline prices [34–36, 40]. Patient opportunity time costs and PHD costs vary depending on whether a patient travels or the PHD sends an outreach worker to deliver medications. See Appendix Section A4.

Overall, about 49% of patients with active TB are hospitalized at least once. Most patients are hospitalized before diagnosis and initiation of antimicrobial therapy; however, 8% of patients require hospitalization while already undergoing treatment [23]. Class B1 NIVs who are diagnosed with active TB during follow-up are expected to begin antimicrobial therapy before developing more acute symptoms leading to a better prognosis and a lower hospitalization rate. We assumed a higher hospitalization rate (49%) for persons diagnosed passively than for those who would be actively detected during recommended follow-up at PHDs (8%) [23, 41]. Hospitalization costs were estimated using the 2006–2011 National Inpatient Survey, and were updated to 2013 dollars [26]. See Appendix Section A4.

Patients with active TB may forgo normal day-to-day activities, such as household and leisure activities, due to illness or infectiousness. We applied an estimate of 12.5 days of disease impairment to calculate time costs based on a previous study [42]. Hospitalized patients were assumed to experience an additional 14 days of disease-related impairment (26.5 days in total) based on the average duration of TB-related hospitalizations [26].

#### Contact tracing

Public health departments use contact investigations to identify individuals who may have been exposed to patients with active TB [43, 44]. For each patient with active TB, PHDs were estimated to spend $164 per contact [43] for an estimated 5.8 contacts diagnosed with TB per case [44].

#### Follow-up visits for persons with class B1 designations

NIVs with clinical findings suggestive of TB in India and Mexico (Class B1) who follow up at local PHDs would incur costs, even if they do not develop active TB. By country of origin, the fraction of immigrants with B1 status who follow up at PHDs and the types of tests performed are reported in the EDN dataset [24, 45]. For persons without active TB, follow-up consultations can vary from a simple exam with no diagnostic tests to a chest radiograph with a set of three sputum smears and cultures (Table 2). We estimated costs of medical care based on the level of testing performed. See Appendix Section A4.

#### Visa and recruitment costs paid by employers

Under the screening program, H-1B employers may incur costs if workers with active TB are prevented from entering the United States. DGMQ TB Indicator data show that 61% of Indian immigrants and 77% of Mexican immigrants were treated at panel physician sites after being diagnosed with active TB. If active TB is treated at a panel physician site, it is assumed that NIV work visa applicants, like immigrant visa applicants, would not have to undergo further medical examination (other than updating the immigration medical examination) after completion of treatment or to reapply for visas. However, if visa applicants seek treatment from a treatment program not under the auspices of the panel physician, they would be inadmissible to the United States for 1 year after successful completion of treatment [4]. In such cases, the H-1B employer may find a replacement [46]. We assumed that the country-specific rates of panel physician treatment would be similar for H-1B applicants as for immigrants, and we assumed potential employers would incur recruitment ($3625) and visa costs ($2000) for additional workers to replace applicants with active TB who would seek treatment at alternative locations. These costs were not estimated for Mexican H-2A or H-2B applicants because of differences in visa fee structures and recruitment practices. See Appendix Section A4.

### TB control activities and associated costs in India and Mexico

#### TB screening, diagnosis, and treatment

Under the screening program, visa applicants must undergo medical examinations performed by panel physicians. Exams include a physical exam, medical history, CXR, and other medical tests [4]. Persons whose medical histories or CXRs suggest TB are required to provide three sputum samples for microscopic smear testing and TB culture. See Table 2.

In India, at the time of this analysis, the panel physician medical exam fee includes the CXR but does not include sputum testing or TB treatment. We applied an estimate from a previous study that 30% of the base panel physician medical exam costs in India are attributable to TB screening [13]. All H-1B visa applicants would incur this cost, but only applicants with clinical findings suggestive of TB would pay for sputum tests.

The fraction of applicants with active TB who would obtain treatment at Indian panel physician sites was estimated from the DGMQ TB Indicator data. Panel physician costs were estimated for these applicants based on cost data provided by Indian panel physicians. Applicant costs to receive treatment at a local healthcare facility incurred were estimated using reported average costs from the World Health Organization (WHO) [47].

In Mexico, panel physician exam fees include TB screening, diagnosis, and treatment as well as screening for other conditions. Thus, all applicants pay the same fee irrespective of TB findings. We estimated that TB-related activities comprise 50% of the exam costs (see Appendix Section A5). Similar to Indian estimates, the fraction of patients treated at panel physician sites was estimated from the TB Indicator data, and cost estimates for treatment at local facilities were based on WHO data [47].

#### Time costs associated with TB screening and treatment

Time costs associated with screening, treatment, and disease-related impairment were computed using country- and occupation-specific wages after adjusting for purchasing power parity (PPP) [27, 28, 30]. Time costs for Indian family members were based on Indian gross domestic product (GDP) adjusted for PPP [29]. See Appendix Section A5.

### Cost-effectiveness model

Each cohort proceeded through the “Screening” and the “No Screening” arms of the decision tree model (see Fig. 1 and Table 1). For “Screening,” costs are incurred for the panel physician medical exams, and there are additional costs if applicants need diagnostic tests or treatment for TB. For “No Screening,” all active TB cases are diagnosed passively, so all costs accrue in the United States.

The primary public health outcome measure is the number of U.S. TB cases under each arm over the course of 1 year. Our final outcome is an incremental cost-effectiveness ratio calculated (ICER) using the following formula [13]: [(“Screening” cost) – (“No Screening” cost)] / [(U.S. TB cases with “No Screening”) – (U.S. TB cases with “Screening”)].

#### Sensitivity analysis

We conducted a number of one-way sensitivity analyses to examine uncertainty in our key model input parameters (see Table 4 and Appendix Section A6). The potential number of U.S. cases averted and multinational cost per U.S. cases were estimated as a function of this correction factor to adjust expected rates of TB in NIVs relative to the rate currently observed for immigrants. A direct application of Indian immigrant prevalent and incident TB rates to the H-1B and H-4 visa applicant population would result in 224 cases. In comparison, 2012–2013 DTBE surveillance data identified about 95 cases annually among all Indians during their first year in the United States [22, 32]. Direct application of immigrant prevalent and incident TB rates to our hypothetical population of Mexican workers would result in an estimate of 52 cases during their first year in the United States, which is more consistent with U.S. TB surveillance data for Mexicans within the first year of arrival into the United States (110 cases on average). In addition, immigration status at arrival was infrequently reported in U.S. TB surveillance data among individuals from Mexico that recently relocated to the United States. See Appendix Sections A3 and A6.

## Results

A TB screening program (“Screening”) for NIV visa applicants in India (H-1B + H-4) and Mexico (H-2A + H-2B) was estimated to avert 179 active TB cases from the United States annually if detection rates in NIVs are consistent with rates observed in the immigrant population. TB-related hospitalizations among these populations in the United States would decrease from 110 to 14 (Table 3). Under the “No Screening” current baseline, 224 NIVs (H-1B and H-4) from India and 52 NIVs (H-2A and H-2B) from Mexico are estimated enter the United States with active TB annually (Table 3).

**Table 3:** Estimated total annual economic costs for tuberculosis screening of nonimmigrant visa applicants, by program and country

	India (***N*** = 102,909)		Mexico (***N*** = 81,439)	
	No screening	Screening	No screening	Screening
**NIV time costs**	$1,924,937	$23,300,164	$135,841	$2,026,805
**NIV out-of-pocket costs**	$31,694	$1,562,287	$4167	$7,756,105
**Hospitalization costs**	$2,414,143	$317,543	$557,442	$38,758
**PHDs’ treatment costs**	$999,064	$383,877	$343,637	$70,249
**PHDs’ follow-up (Class B1)**		$492,898	–	$259,781
**Employer-forfeited visa/recruitment costs**		$189,118	–	$0
**Total costs (U.S. and international)**	$5,369,838	$26,245,885	$1,041,087	$10,151,698
**Total U.S. costs**	$5,369,838	$2,213,938	$1,041,087	$442,350
**Net** multinational **costs (U.S. and international)**		$20,876,047		$9,110,611
**Cost savings to the U.S.**		$3,155,900		$598,737
**# of U.S. TB cases**	224	86	52	11
**# of hospitalizations**	110	14	25	2
**Incremental cost-effectiveness ratio**		$151,388		$221,088

Notes: *NIV* Non-immigrant visa applicants, *PHDs* Public health departments, *U.S.* United States

Out-of-pocket costs include worker transportation and forfeited visa fees paid by applicants in the United States, and panel physician exam fees paid by applicants in their home countries

Class B1 indicates persons who have an abnormal chest radiograph during panel physician screening exams

ICER = Incremental Cost-effectiveness Ratio (computed by dividing the difference in costs by the difference in effects)

Totals may not sum up exactly because of rounding effects

Under “No Screening,” the multinational costs associated with treating TB for NIVs from India are approximately $5,369,838 (Table 3). The majority of costs are due to U.S. hospitalization (45%) and patient opportunity costs (36%) (i.e., costs for time spent on screening, diagnosis, and treatment-related activities). PHDs incur about 19% of the multinational costs for treatment. NIVs’ out-of-pocket costs for travel are estimated to be 0.6%.

For Indian NIVs, multinational costs under “Screening” would increase to $26,245,885 (Table 3). Workers’ and family members’ opportunity costs (89%) and out-of-pocket expenses (6%) would comprise 95% of the total costs. Costs to U.S. PHDs for TB treatment and follow-up for Class B1 NIVs, and employer costs for recruitment and forfeited visa fees would together comprise about 4% of the multinational costs for “Screening.”

For Mexican workers, the multinational costs under “No Screening” are $1,041,087 (Table 3). The majority of costs are for TB-related hospitalizations (54%). PHDs absorb about 33% of total costs for TB treatment. NIVs’ opportunity costs comprise about 13% of total costs.

Under “Screening” for Mexican NIVs, the multinational costs would increase to $10,151,698 (Table 3). NIVs’ out-of-pocket (76%) and opportunity (20%) costs would be the primary cost components. U.S. PHDs would incur about 1% of total costs for treatment of TB and 3% of total costs for following up with Class B1 NIVs. Hospitalization costs would be < 1%.

From the U.S. perspective, “Screening” would result in cost savings (Table 3). Hospitalization costs would decrease by $2,096,600 for Indian NIVs and $518,684 for Mexican NIVs. PHD costs would decline by an estimated $122,290 for Indian NIVs and $13,607 for Mexican NIVs. In comparison, opportunity and out-of-pocket costs would increase by $22.9 million for Indian NIVs and by $9.8 million for Mexican NIVs.

The multinational costs per U.S. case averted (ICERs) were $151,388 for NIVs from India and $221,088 for NIVs from Mexico (Table 3).

**Fig. 2:**
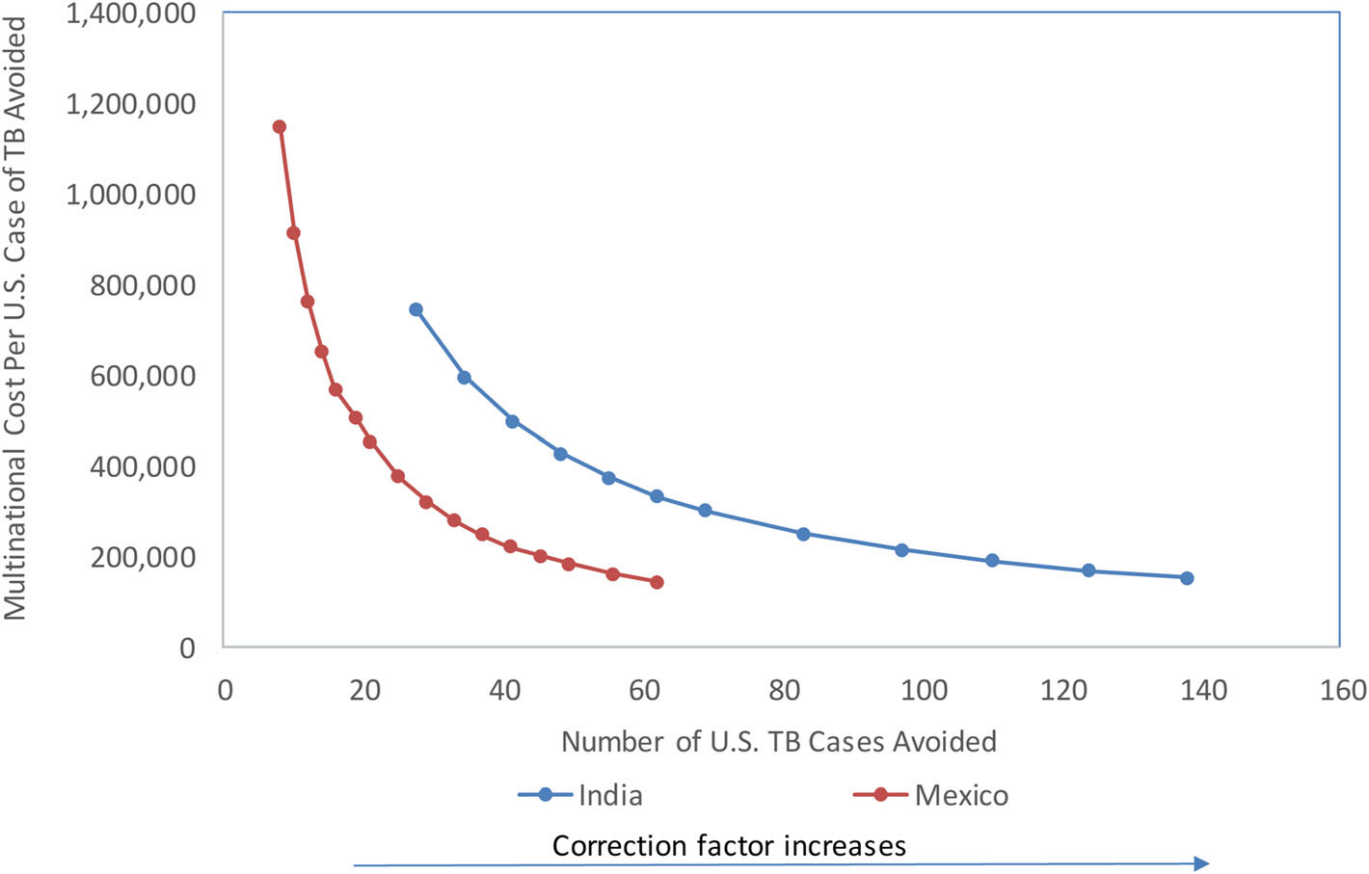
Cost per U.S. Case of Tuberculosis Avoided Under a Screening Program for Indian H-1B and H-4 Nonimmigrant Visa Applicants. Notes: Graph presents the cost per U.S. case of tuberculosis (TB) avoided as the number of U.S. TB cases avoided and correction factor increases. The correction factor was varied between 10 and 100% to account for the potential differences between immigrants and NIVs. In the model, the screening outcomes for immigrants are applied to the NIV population; however, it is possible that the TB prevalence would be lower in the NIV population. The correction factor was applied to the percent of the applicants with evidence of TB (resulting in Class B1 NIV arrivals recommended for U.S. follow-up and cases diagnosed pre-departure). NIV = Non-immigrant visa applicant. H-1B = type of visa for highly skilled workers. H-4 = type of visa from family members of H-1B visa holders

We conducted a variety of sensitivity analyses to determine how varying key parameters changes the multinational ICERs (Table 4). The most important source of uncertainty for the Indian NIV population is the correction factor for the proportion with clinical findings suggestive of TB. Correction factors ranging from 20 to 100% resulted in ICERs varying from $1,484,067 to $151,388, and the number of cases avoided varying from 14 to 138 (Fig. 2). For Mexico, varying the proportion with clinical findings suggestive of TB by 20 to 150% resulted in ICERs ranging from $1,147,596 to $143,879. Varying the proportion of Class B1 NIVs diagnosed with TB after entering the United States by 50 to 150% had a smaller effect and resulted in ICERS ranging from $154,135 to $148,641 for India and $222,075 to $220,100 for Mexico. The effect of varying the time spent on screening and treatment at panel physician sites by 66 to 166% resulted in ICERs ranging from $42,654 to $260,665 for India and $220,644 to $221,532 for Mexico.

**Table 4:** One-way sensitivity analyses of the costs of tuberculosis screening of nonimmigrant visa applicants: cost per estimated case averted by varying key parameters, by country

	India			Mexico		
	Estimated U.S. cases prevented before U.S. arrival	U.S. Savings	ICER	Estimated U.S. cases prevented before U.S. arrival	U.S. savings	ICER
Base case	138	$3,155,900	$151,388	41	$598,737	$221,088
Proportion with clinical findings suggestive of TB^a^ (decreased by 50%)	69	1,577,950	299,463	21	$299,369	$452,715
Proportion with clinical findings suggestive of TB^a^ (increased by 50%)	NA^b^	NA	NA	62	$898,106	$143,879
Proportion of B1 persons diagnosed domestically (reduced by 50%)	138	$2,777,095	$154,135	41	$558,045	$222,075
Proportion of B1 persons diagnosed domestically (increased by 50%)	138	$3,534,706	$148,641	41	$639,430	$220,100
Time spent on panel physician treatment in India or Mexico (reduced by 67% of baseline)	138	$3,155,900	$42,654	41	$598,737	$189,459
Time spent on panel physician treatment in India or Mexico (increased by 67% of baseline)	138	$3,155,900	$260,665	41	$598,737	$252,875
Opportunity costs excluded	138	$499,180	Cost saving	41	$528,421	$175,200

Notes: *ICER* Incremental Cost-effectiveness Ratio, *U.S.* United States

^a^In the model, individuals without clinical findings suggestive of TB do not go on to sputum culture/smear testing. If we assume that same rates of diagnosis (active TB) both overseas and at domestic follow-up for immigrants and NIVs, then this single parameter affects both the number of TB cases diagnosed overseas and the number of B1s. See Appendix Section A3 for details

^b^We did not evaluate a 50% increase for Indian NIVs because this did not seem plausible given

## Discussion

Similar to a previous study [13], we find that screening NIVs with work visas or their family members would result in cost savings and reduction in active TB cases among persons from India and Mexico from the U.S. perspective. From a multinational perspective, the cost per U.S. case avoided increases as the prevalence in the populations screened decreases, e.g., the expected number of cases detected per 100,000 Indian NIVs screened is 134 compared to about 50 for Mexican NIVs. The additional screening costs would accrue to NIVs in the form of increased opportunity costs and out-of-pocket costs.

Skilled Indian NIVs with H-1B visas receive substantially higher wages than their lower-skilled Mexican counterparts with H-2A and H-2B visas. For Indian NIVs, opportunity costs would increase from $18.71 per person under “No Screening” to $226.42 per person under “Screening” after adjusting for PPP. These opportunity costs comprise most of the multinational societal costs for Indian NIVs. For Mexican NIVs, the difference is not quite as high, from $1.67 per person under “No Screening” to $24.89 per person under “Screening.” Opportunity costs are important because there are a limited number of panel physicians in each country; we assumed screening would take 1 full day, even for persons with no evidence of TB. In contrast, Mexican NIVs’ out-of-pocket costs are higher than Indian NIVs’ due to the differences in panel physician fees ($95 in Mexico and $26 in India on average).

Under the current baseline, NIVs’ out-of-pocket costs were limited to transportation-related costs in the United States because PHDs absorb most of the diagnosis- and treatment-related costs and we were unable to estimate the NIVs’ payments/copayments for hospitalization costs.

From the U.S. perspective, hospitalization and PHD treatment costs would decline under “Screening” because there would be fewer active U.S. TB cases. Moreover, most of the U.S. cases would be diagnosed actively, resulting in a lower likelihood of hospitalization. Although PHDs would incur additional costs associated with follow-up visits from Class B1 NIVs, we estimate that such costs could be offset by the treatment cost savings from cases treated in NIVs’ home countries.

Employers recruiting skilled NIVs from India would incur costs in the form of forfeited visa fees and recruitment expenses if they cannot wait for visa applicants to be treated or if applicants with active TB elect to receive treatment from a local healthcare site.

Limitations associated with the study may affect our findings. First, we assume that NIVs have TB case rates similar to those of immigrants. This assumption may not be reasonable for Indian NIVs because they are employed in high-paying positions. If Indian NIVs have higher socioeconomic status than immigrants, their TB rate may be lower. By comparison, U.S. passive surveillance data from 2012 to 2013 included an average of 514 TB cases among all individuals born in India and an average of 95 cases in those who had arrived in the previous year. This compares with our model estimates of 138 cases diagnosed during NIV screening in India (corresponding to a rate of 134 cases per 100,000 screened) and another 86 cases diagnosed after arrival based on application of Indian immigrant data to the NIV population. In comparison, there were an average of 1276 cases per year diagnosed among individuals born in Mexico. Of these, an average of 110 cases per year were diagnosed among individuals who had arrived in the United States during the previous year [32]. From the model, we estimate that 56 cases per year would be diagnosed among Mexican NIVs prior to U.S. arrival and another 14 cases diagnosed after arrival. These estimates are less than the 110 reported cases per year diagnosed among individuals born in Mexico during their first year in the United States. However, about 25% of tuberculosis patients from Mexico and Latin America self-reported undocumented status [10], so the two numbers are not directly comparable.

The length of time between when a case is diagnosed as culture-positive based on active screening versus when the same patient would have developed symptoms and seek treatment in the absence of such screening is not known. An analysis of a decline in the incidence of non-U.S.-born individuals diagnosed with TB after arrival was undertaken around the same time that CDC changed its requirements for immigrant and refugee pre-arrival TB screening. In 2007, panel physicians trained to implement medical examinations for U.S.-bound immigrants began to switch from microscopic smear-based sputum testing to a combination of smear and culture sputum testing. The addition of cultures increased the number of individuals diagnosed with infectious TB during pre-departure screening and to reduce the number of immigrant and refugee visa holders diagnosed with infectious TB after U.S. arrival [45, 48, 49]. Baker et al. reported that the non-U.S.-born tuberculosis case detection rate in the United States decreased by 30% each year during the first 3 years after arrival between 2007 and 2011, but only by about 8.5% for individuals who had arrived between 3 and 6 years prior to being diagnosed with infectious TB. This suggests that active screening and early detection might reduce case detection rates for the non-U.S.-born population for more than a year after arrival; however, the authors were careful to note that the change in immigrant screening protocols was unlikely to be the sole driver in the decline in TB case detection rates among newly-arrived individuals in the non-U.S.-born population during this period [48].

We conducted sensitivity analysis by examining how NIV rates equivalent to 20 to 100% of immigrant rates would affect the numbers of cases detected and multinational cost per case averted. However, it is difficult to compare the outcomes from active screening with passive surveillance data. The screening activities may result in earlier diagnosis (i.e. more than 1 year after arrival) or detection of cases that would not have been identified until after NIVs returned to their home countries. In contrast, Mexican NIVs (agricultural and seasonal workers) may have lower socioeconomic status than their immigrant counterparts. TB rates are typically higher among those in lower-income groups, so our findings are probably conservative estimates of the number of U.S. active TB cases avoided from individuals with work visas from Mexico [50, 51].

By way of comparison, both Australia [52] and the United Kingdom (UK) [53] already screen long term non-immigrant visitors including workers and students from high-incidence countries. Australia and the UK respectively reported case detection rates of 53 and ~ 270 cases per 100,000 screened Indian visa candidates across all categories. Our estimated case detection rate for India was 138 per 100,000 NIV candidates, which falls within this range. Neither Australia nor the UK reported rates for Mexican visa candidates for comparison.

Our results underestimate TB treatment costs because we did not account for treating multidrug-resistant TB, disease transmission, or the costs resulting from premature TB-related death [54–56]. On average, about 1.8% of non-U.S.-born TB patients have multidrug resistant tuberculosis [57]. A recent publication by Castro et al. estimated that the average societal cost per U.S. drug-susceptible TB case was $20,000 in 2014 U.S. dollars, inclusive of productivity costs [54]. This is very similar to our estimate of cost per TB case for Mexican NIVs ($20,133) and slightly lower than our estimate for Indian NIVs ($23,978). The higher estimate for Indian NIVs is probably due to the higher opportunity cost of time used for Indian NIVs, who are assumed to have high-paying jobs. The recent publication also reports societal costs per multidrug resistant TB case ($225,000) and per extremely drug-resistant TB case ($621,000). If we assume that 1.8% of cases among non-U.S.-born NIVs are multidrug resistant, the weighted average cost per case estimate would be $23,690. All of the above estimates do not include the productivity costs associated with premature death, which were estimated to increase the cost per drug-susceptible case from $20,000 to $44,000 and per multidrug resistant case from $225,000 to $282,000 according to Castro et al. The inclusion of these costs would have a much greater impact on the estimated costs of untreated TB in the U.S. as the weighted average cost per case (assuming 1.8% of cases are multidrug resistant) would be $48,284 or more than double the estimate without consideration of premature mortality in our analysis ($20,133 to $23,690). A higher cost estimate for passively detected cases would increase the cost of the current “No Screening” baseline and improve the cost effectiveness of the “Screening” alternative.

Further, some of our assumptions to subdivide costs by stakeholder may oversimplify how workers would pursue treatment or who would incur treatment or panel physician exam costs. For example, we assumed that all TB-related outpatient costs will be borne by PHDs, but some NIVs may seek treatment from private providers, and their insurance may cover some outpatient treatment costs. However, these assumptions would lead to more conservative estimates of U.S. cost savings from screening NIVs in countries of origin; actual cost savings may be even greater.

Finally, NIVs with clinical findings suggestive of TB, but negative culture and smear results from overseas screening (Class B1), may also be evaluated for latent tuberculosis infections if they follow-up with local health departments after arrival as recommended. Individuals found to have latent infection may be offered treatment to reduce their risk of developing infectious tuberculosis. This could further reduce the burden of tuberculosis in these populations in subsequent years. However, the costs and future health impact of treatment for latent tuberculosis infection was not analyzed since the hypothetical program for NIVs is based on testing and treatment for infectious tuberculosis rather than latent infection.

## Conclusions

TB screening for NIVs from India and Mexico would prevent up to an estimated 179 U.S. active TB cases and result in substantial cost savings ($3,754,638) to the United States annually. The majority of multinational costs for screening programs would be borne by workers seeking visas to the United States. At the same time, mandatory TB screening for NIVs would increase out-of-pocket payments and time costs for NIVs to undergo screening.

TB elimination is a priority in the United States [58]. Currently, about 65% of TB cases occur among the non-U.S.-born population. Broadening the screening program to include NIVs would identify individuals with TB in their home countries so they could obtain treatment before entering the United States. Such a program could reduce the burden of TB among non-U.S.-born individuals in the United States and assist in achieving U.S. TB elimination goals.

## Supplementary Information

**Additional file 1.**

## Data Availability

The authors would be happy to include the Excel and Treeage analysis files as supplemental information with this submission.

## Abbreviations

B1: Class B1 refers to immigrants who have clinical findings suggestive of TB, but whose sputum samples are not culture-positive or smear-positive.
CDC: U.S. Centers for Disease Control and Prevention
CXR: Chest radiograph
DGMQ: CDC’s Division of Global Migration and Quarantine
DOT: Directly observed therapy
DTBE: CDC’s Division of Tuberculosis Elimination
EDN: Electronic Disease Notification database
GDP: Gross domestic product
ICER: Incremental cost effectiveness ratio
NIV: Nonimmigrant visa applicants
PHD: Public health department
PPP: Purchasing power parity
SAT: Self-administered therapy
TB: Tuberculosis
TI: Technical Instuctions
TB TI: Tuberculosis Technical Instructions
UK: United Kingdom
U.S.: United States
WHO: World Health Organization
