# Supplementary material for: Cost effectiveness analysis of implementing tuberculosis screening among applicants for non-immigrant U.S. work visas
Source: Pneumonia (Nathan). 2020 Dec 25;12:15. doi: 10.1186/s41479-020-00078-z (PMC7761151; doi:10.1186/s41479-020-00078-z)
Supplement: Supplementary file 1 — Additional file 1. [file 41479_2020_78_MOESM1_ESM.docx]

**Cost Effectiveness Analysis of Implementing Tuberculosis Screening Among Applicants for Non-immigrant U.S. Work Visas**

**Appendix**

Bisma Ali Sayed, PhD;^a^ Drew L. Posey, MD, MPH;^a^  Brian Maskery, PhD;^a^ La’Marcus T. Wingate, PharmD, PhD;^b^ Martin S. Cetron, MD,^a^

^a^-Division of Global Migration and Quarantine; Centers for Disease Control and Prevention; Atlanta, GA

^b^-College of Pharmacy; Howard University; Washington DC

# A1. Tuberculosis Technical Instructions (TB TI) for Screening and Treatment of Visa Applicants

Under authorities defined by the Immigration and Nationality Act (INA) and the Public Health Service Act, the Secretary of Health and Human Services promulgates regulations outlining the requirements for medical examinations of aliens seeking admission into the United States (1) The Public Health Service Act authorizes the Centers for Disease Control and Prevention (CDC) to prevent the introduction, transmission, and spread of specific communicable diseases in the United States, including infectious tuberculosis (2). CDC’s Division of Global Migration and Quarantine has developed requirements for tuberculosis screening and treatment that must take place in applicants’ home countries. These requirements are outlined in the Tuberculosis Screening and Treatment Technical Instructions (TB TIs) (3). At present, only candidates for immigrant and refugee visas are routinely required to undergo medical exams as part of the visa application process.

If TB risk factors are identified (e.g., abnormal chest radiograph), persons are required to submit three sputum samples for smears and cultures (3). If smears and cultures indicate no active disease, persons are given a Class B1 visa classification and are advised to follow up with local public health departments after arrival into the United States (3). If active infectious TB disease is present, persons receive a Class A visa classification and are required to complete treatment as specified in the TB TIs before they can enter the United States (3).

For immigrants and refugees to be eligible to enter the United States within 6 months, they must complete treatment at an approved panel physician site. If treatment is not obtained at an approved panel physician site, persons are ineligible to reapply for 1 year, after which they must resubmit their visa application and undergo a medical examination.

# A2. Tuberculosis Rates in India and Mexico

According to the World Health Organization (WHO), India is a high TB burden country, with a national annual incidence rate of 171 per 100,000, and a prevalence rate of 211 per 100,000 [4]. Mexico is a moderate burden TB country, with a national annual incidence rate of 21 per 100,000, and a prevalence rate of 26 per 100,000 population (4).

# A3. Target Population and Estimated Tuberculosis Cases

We constructed a hypothetical cohort for each country based on average noniimigrant visa issuances for selected categories from each country from 2010 to 2012.

**Table A3.1 Number of H-1B and H-4 Visas Issued in India and H-2A and H-2B Visas Issued in Mexico by Year, 2010-2012**

|  | **India** | | **Mexico** | |
| --- | --- | --- | --- | --- |
| **Year** | **H-1B** | **H-4** | **H-2A** | **H-2B** |
| 2010 | 58,664 | 38,833 | 52,317 | 33,375 |
| 2011 | 72,438 | 46,969 | 51,927 | 36,179 |
| 2012 | 80,630 | 53,877 | 61,324 | 36,341 |
| Average | 70,577 | 46,560 | 55,189 | 35,298 |
| Final Cohort Size* | 63,520 | 39,389 | 49,670 | 31,769 |

H-1B visas: specialty occupations in fields requiring highly specialized knowledge

H-2A visas: Temporary agricultural worker.

H-2B visas: Temporary worker performing other services or labor of a temporary or seasonal nature

H-4 visas: Immediate family members (spouse or children) of H-1B visa holders

* The Office of Immigration Statistics reports the total number of temporary workers and families entering the United States based on I-94 forms (5). Among persons issued H-4 visas, data indicate that 94% are adults. We applied this percentage to all Indian H-4 visa applicants to estimate the number of adults that would accompany H-1B visa holders. To compute final cohort size, we assumed that 10% of persons issued visas will not enter the United States.

Source: U.S. Department of State, Bureau of Consular Affairs (6) and Office of Immigration Statistics (5)

To estimate the number of active TB cases in each cohort, we used the DGMQ Tuberculosis Indicator Data. These data are maintained by panel physicians to report aggregate screening outcomes to DGMQ. We estimated the proportion of persons with clinical findings suggestive of TB (i.e. abnormal chest radiographs [CXR] or other signs and symptoms of TB), and among these, the proportion who have active TB for each country. These country-specific estimates were applied to our hypothetical cohorts to estimate the number of persons who would be given a Class B1 visa classification and the number of active TB cases diagnosed in each country by panel physicians.

**Table A3.2 Panel Physician Screening Exam Tuberculosis Outcomes for Immigrants or Refugees in India and Mexico (2012-2013), by Country**

| **Country** | **# of Applicants Screened (X)** | **Proportion with CXR Suggestive of Tuberculosis (Y)** | **Proportion with Active Tuberculosis Among Those with Abnormal Chest Radiographs (Z)** | **Case detection rate per 100,000 Applicants (Y *Z * 100,000)** |
| --- | --- | --- | --- | --- |
| India | 43,042 | 0.0335 | 0.04 | 134 per 100,000 |
| Mexico | 171,921 | 0.022 | 0.02 | 44 per 100,000 |

Notes: Proportion with active tuberculosis is based on the number of persons with chest radiographs (CXR) suggestive of tuberculosis.

Source: DGMQ Tuberculosis Indicator Data

For Indian workers and their families, we conducted a sensitivity analysis to account for the possibility that individuals receiving H-1B or H4 visas may not be as likely to have active TB disease as the population receiving immigrant visas and for which data were available. For the sensitivity analysis, we incorporated a correction factor to the probability of immigrants’ having abnormal CXRs or other signs and symptoms of TB. This correction factor affects the number of TB cases diagnosed pre-departure and the number of individuals arriving in the United States with class B1 status. Based on existing national surveillance data in the United States, we calculated available active TB cases reported in U.S. surveillance data (7, 8). We followed the steps below:

1. Estimated the annual average number of active TB cases in a 2-year period that occur among persons from India who have been in the United States for 1 year or less (7, 8).
2. Examined the average annual number of active TB cases in a 2-year period by immigration status. During the 2-year period 2012-2013, an average of 10 cases of TB occurred per year among persons who self-reported entering the United States with an employment visa. This variable contained missing cases and it is possible that people misreported their immigration status. There were also 38 persons in 2012 and 45 persons in 2013 from India diagnosed with TB who did not have any information on their length of time in the U.S. before diagnosis. We assumed that some of these cases with unknown duration in the United States would have occurred during the first year. According to data from the CDC Division of Tuberculosis Elimination, among reported cases with known duration in the United States, 17% of cases among Indians in 2012 occurred in the first year post U.S. arrival and 20% of cases among Indians in 2013 occurred in the first year post U.S. arrival. We applied these percentages to the number of cases for which the number of years in the United States was not reported to estimate how many of these cases would occur during the first year after arrival into the United States. See Table A3.3.

Based on the above methodology, about 15.5 cases per year were observed among individuals from India who reported arriving with an employment or family visa. In addition, there were about 45 cases per year among individuals with no self-reported visa-at-arrival information available and an estimated 7.7 cases per year may occur among individuals for whom the number of years in the United States was not reported.

Since the visa type for persons with TB is reported sporadically in U.S. surveillance data, the number estimated in our target population cannot be compared directly (i.e., cases could occur in immigrants, students, diplomats, or other populations not included in our hypothetical cohorts). In addition, an active screening program may expedite the diagnosis of cases (i.e. cases diagnosed passively in the second year after arrival may instead be diagnosed during active screening depending on the pace of disease progression).

The rate in immigrants was consistent with data reported in the CDC EDN System database for immigrants with Class B1 status (9). This suggests that the predeparture exam is effective in both diagnosing cases to be treated prior to arrival and flagging immigrants at elevated risk to develop active TB after arrival.

| **Table A3.3 Reported TB Cases among Individuals Born in India During their First Year after Arrival in the United States by Immigration Status as Reported in the RVCT (2012-2013) (7, 8)** | | |
| --- | --- | --- |
| Immigration Status | TB Cases in 2012 and 2013 | Average for a 1-year Period |
| Asylum | 1 | 0.5 |
| Employment visa | 20 | 10 |
| Family visa | 11 | 5.5 |
| Immigration visa | 30 | 15 |
| Refugee | 0 | 0 |
| Student visa | 16 | 8 |
| Tourist visa | 20 | 10 |
| Missing (immigration status) | 90 | 45 |
| Additional estimated cases | 15 | 7.7 |
| Total | 204 | 102 |
| Notes: RVCT = Report of Verified Case of Tuberculosis (TB). Data are for verified cases of TB for the years 2012-2013 in persons who had been in the United States for 1 year or less, among people reporting their home country was India (7, 8). Additional cases were estimated by taking the total number of cases with unknown duration in the United States for each year and assuming that a fraction would occur in the first year.  Note that equivalent data for individuals born in Mexico was not presented because immigration at arrival data were unavailable for most individuals with infectious TB diagnosed in the United States. | | |

Note that we were unable to do a similar comparison for individuals from Mexico, because immigration status was less frequently reported among TB patients that reported arriving from Mexico within the previous year. For Mexican nonimmigrant visa applicants (NIVs), we included a wider uncertainty range (20% to 150%) for the sensitivity analysis. We expanded the upper bound to 150% to account for the possibility that individuals applying for short term work visas may be more likely than Mexican immigrant visa candidates to have active TB.

To estimate the number of annual incident cases that would be diagnosed within 1 year after arrival into the United States, we used CDC’s Electronic Disease Notification System. We applied these country-specific proportions to our hypothetical cohorts to estimate the number of active TB cases that would occur in the United States for each country. We found that 2.6% of Class B1 Indian and 0.6% of Class B1 Mexican immigrants and refugees were diagnosed with TB at follow-up exams. We again assumed that these proportions would be similar for immigrant and non-immigrant visa candidates.

| **Table A3.4 Tuberculosis Follow-up Outcomes of US Immigrant Arrivals from India with Class B1 Status, 2011-2014** | | | |
| --- | --- | --- | --- |
|  | Diagnosis | # of Persons | Percentage of Total |
|  | No TB exposure | 304 | 28.2 |
|  | Exposure to TB but not latent TB infection | 65 | 6.0 |
|  | Latent TB infection | 304 | 28.2 |
|  | Active TB disease | 28 | 2.6 |
|  | Old, healed, inactive TB disease | 378 | 35.0 |
|  | Total | 1079 | 100 |

Notes: Data are for immigrants and refugees from India who are 15 years and older and arrived with Class B1 status from the panel physician medical exam. Data are from years 2011 to 2014. All persons with missing follow-up outcomes were excluded from the analysis.

TB = Tuberculosis

Source: Electronic Disease Notification System (9)

| **Table A3.5 Tuberculosis Follow-up Outcomes of US Immigrant Arrivals from Mexico with Class B1 Status, 2011-2014** | | | |
| --- | --- | --- | --- |
|  | Diagnosis | # of Persons | Percentage of Total |
|  | No TB exposure | 1,407 | 45.2 |
|  | Exposure to TB but not latent TB infection | 220 | 7.1 |
|  | Latent TB infection | 1,022 | 32.8 |
|  | Active TB disease | 19 | 0.6 |
|  | Old, healed, inactive TB disease | 446 | 14.3 |
|  | Total | 3,114 | 100 |

Notes: Data is for immigrants and refugees from Mexico who are 15 years and older and received a Class B1 designation on the panel physician medical exam. Data is from years 2011 to 2014. All persons with missing follow-up outcomes were excluded from the analysis.

TB = Tuberculosis

Source: Electronic Disease Notification System (9)

# A4. Domestic Processes and Costs

*Domestic Opportunity Costs*

India

U.S. Citizenship and Immigration Services reports that the majority of H-1B workers are employed in computer-related occupations [9]. Within this broad category, a large share of workers are employed as system analysts (10). We used this occupation to value opportunity costs. We obtained the median annual salary ($79,680, OCC-CODE 15-1121) from the Bureau of Labor Statistics (BLS), updated to 2013 dollars ($80,847) to estimate time costs for Indian H--1B workers (11, 12). Salaries were adjusted for benefits (30%). For adult family members of H-1B visa applicants, we used GDP per capita per hour in 2013, adjusted for purchasing power parity (PPP), to value time costs (13). The GDP per capita adjusted for PPP was $53,042, which translates to $6.06 per hour, which was applied to estimate the opportunity costs for H4 visa candidates (13).

Mexico

For Mexican H-2A workers, we used salaries of farmworkers in the United States. According to the BLS (OCC-CODE 45-2090), agricultural workers earned $9.09 per hour in 2012 (12). We updated this to 2013 dollars using the U.S. Consumer Price Index ($9.22) (13).

To estimate the opportunity costs for Mexican H-2B workers, we calculated the average wages across the top five occupations that are issued foreign labor certificates in Mexico, but we reduced this figure by 20% because these workers often make less than their U.S. counterparts. The top five occupations that are issued foreign labor certificates are listed in Table 5.

**Table A4.1 2013 Annual and Hourly Wages for the Top Five Occupations for Mexican H-2B (Seasonal) Workers, Estimates from the Bureau of Labor Statistics, Occupational Employment Statistics (12)**

| **Occupation** | **Mean Annual Wage, 2013** | **Mean Hourly Wage, 2013** |
| --- | --- | --- |
| Landscaping and Groundskeeping Workers (OCC-CODE 45-3011) | $26,293 | $12.64 |
| Forest and Conservation Workers (OCC-CODE 45-4011) | $29,167 | $14.02 |
| Amusement and Recreation Attendants (OCC-CODE 39-3091) | $20,261 | $9.74 |
| Maids and Housekeeping Cleaners (OCC-CODE 37-2012) | $22,140 | $10.64 |
| Meat, Poultry, and Fish Catchers and Trimmers (OCC-CODE 51-3022) | $23,805 | $11.63 |
| **Average Annual Wage** | **$24,333** | **$11.70** |

Source: Bureau of Labor Statistics and Office of Foreign Labor Statistics (11, 12)

We also adjust wages for benefits (30%).

*Diagnostic and Follow-up Visits*

Following previous research and expert opinion, we estimate that each person with active TB receives three diagnostic visits, consisting of one chest radiograph, three sets of smears and cultures, four drug susceptibility tests (i.e. one test for each of four drugs), liver panel tests, and a complete blood count test (14, 15). Follow-up visits include medical tests that altogether consist of the following: two sets of smears and cultures, two chest radiographs, two complete blood cell counts, and two liver panel tests (15). Although individuals may also be tested for TB infection (e.g., with tuberculin skin tests or Interferon Gamma Release Assay tests), we did not include the costs of those tests because we did not examine the potential costs and benefits of diagnosing and treating latent TB in this analysis. This omission may lead to underestimation of the costs of domestic follow-up and future savings for averted TB treatment.

**Table A4.2 U.S. Diagnostic and Follow-up Visits, Quantity and Cost**

| **Components** | **Diagnostic Visits^1^ (Quantity)** | **Follow-up Visits^1^ (Quantity)** | **Cost in USD (2013)** |
| --- | --- | --- | --- |
| **Medical Components** |  |  |  |
| Chest radiograph | 1 | 2 | $35.82 |
| Sputum smears | 3 | 2 | $9.99 |
| Cultures | 3 | 2 | $20.07 |
| Complete CBC with diff | 1 | 2 | $7.93 |
| Serum chemistry | 1 | 0 | $15.71 |
| Drug susceptibility tests | 4 | 0 | $10.50 |
| Hepatic function panel | 1 | 2 | $15.17 |
| **Services** |  |  |  |
| Nurse time | 3 hours | 2.5 hours | 1 hour = $47.77 |
| Physician time | 1.5 hours | 1 hour | 1 hour = $127.57 |
|  |  |  |  |

Each person with active tuberculosis was assumed to receive three diagnostic visits and five follow-up visits. The costs for medical components were computed using the 2013 Physician Fee Schedule from the Centers for Medicare and Medicaid Services (16), and healthcare worker service time was valued using wage information from the Bureau of Labor Statistics OCC_CODES 29-1141, 29-1062, 29-1061 (12).

^1^Persons with Class B1 TB designation would not receive all diagnostic and follow-up visits unless they were diagnosed with active tuberculosis.

Medical Tests

Cost data for all medical tests were obtained from the Medicare Clinical Fee Schedule 2013 (16).

Personnel

Because public health department cost estimates for treatment of active TB were unavailable, we estimated approximately 30 minutes of physician time and 1 hour of nurse time for each diagnostic visit (14, 15). For each follow-up visit, we estimated 12 minutes of physician time and 30 minutes of nurse time. Table A4.3 presents annual wages for healthcare occupations.

**Table A4.3 Healthcare Occupation Annual Wages**

| **Healthcare Occupation** | **Mean Annual Wage, 2013** |
| --- | --- |
| Nurses | $68,676 |
| Physicians | $183,346 |
| Licensed practical and vocational nurses (outreach workers) | $42,727 |

Source: Bureau of Labor Statistics OCC_CODES 29-1141, 29-1062, 29-1061 (12)

All salaries were adjusted for benefits (30%).

We also added 30% of public health staff salaries for non-specified overhead to estimate the total cost for diagnostic and follow-up visits.

Patient Time Value

Following previous research and expert opinion, we estimated 2 hours of patient time for each diagnostic visit and 1.5 hours of patient time for follow-up visits [21, 25]. For Mexican agricultural workers, we added an extra 30 minutes because these workers typically live in areas farther from health facilities [26].

Travel

Travel costs to and from public health departments (PHDs) were estimated based on fuel costs and average mileage to healthcare facilities. The National Household Travel Survey (NHTS) finds that respondents live about 10.58 miles (one way) from a healthcare facility [27]. For diagnostic and follow-up visits, we assumed that patients travel 21.16 miles for each PHD visit. Fuel costs were estimated based on the average fuel price for 2013 ($3.59) and average miles per gallon (24.1) [28, 29].

Treatment

The most common treatment regimen for active TB disease lasts for 6 months and comprises two phases: the initial phase, an intense phase of treatment that lasts for 2 months and requires patients to take four medications (rifampin, isoniazid, ethambutol, and pyrazinamide), and the continuation phase, a less intense phase that lasts for 4 months and includes two medications (rifampin and isoniazid) [3]. We estimated medication costs for a 150-pound person for each phase of treatment using the price per pill reported in the Red Book database [30]. These costs were estimated based on the assumption that TB cases would be susceptible to the standard treatment regimen. A fraction of TB cases may be resistant to the standard treatment regimen; multidrug-resistant TB requires a more expensive and time-consuming drug regimen.

Under directly observed therapy (DOT), PHDs may send a nurse or healthcare outreach worker to patients’ homes to deliver and observe consumption of medications. Alternatively, patients can travel to the health department for DOT. Although more expensive and time-consuming than self-administered therapy (SAT), DOT regimens have better treatment outcomes (17).

Existing data suggest that approximately 62% of persons with TB in the United States were provided with DOT for the entire treatment regimen, 29.3% were provided with a combination of DOT and SAT for the entire treatment regimen, and the remaining self-administered their medication (8). Expert opinion and the TB Wonder data indicated that these figures are not the same for agricultural workers, who often work in remote locations and would have challenges traveling to health departments (18, 19). Thus, we estimated that 62% of agricultural workers were provided DOT, 35% were provided a combination of DOT and SAT, and 3% were exclusively on SAT. We applied these DOT/SAT percentages to cases treated in the United States (Figure 4.1).

**Figure 4.1. Tuberculosis Treatment in the United States**

Travel costs for healthcare workers were estimated based on national reimbursement rates and average mileage to health departments as reported in NHTS. We assumed that health department staff would visit more than one patient during a trip, so we reduced the estimated mileage (21 miles) by half. For agricultural workers from Mexico, we increased the average mileage traveled by healthcare workers to 21 miles based on existing literature (20, 21).

Following previous research, we assumed that 60% of patients would receive visits from a healthcare outreach worker and 40% of patients would travel to the health department for DOT. For patients who traveled to the health department, we used mileage and gas costs to estimate travel-related expenses. For Mexican agricultural workers, we attributed all travel-related costs for treatment administration to the health department, assuming that no patient would travel to the health department for DOT (18).

For Indian NIVs (skilled workers and their adult family members) and Mexican seasonal worker NIVs (H-2B), following previous research, we assumed that if the health department sent an outreach worker to the patient, the patient would incur 8 minutes for medication administration (15). If the patient traveled to the health department, the patient would incur 1 hour. We did not include any costs if the patient self-administered therapy. For Mexico’s agricultural worker NIVs (H-2A), we only included 8 minutes of patient time because the health department would send outreach workers to all patients on DOT to administer medications.

*Follow-up Visits for Persons with Class B1 Designations*

Existing research suggests that 78.6% of all Class B1 immigrants follow up at public health departments (22). We applied this proportion to determine the number of NIVs with B1 status in our cohorts who would present for followup at public health departments.

We relied on Electronic Disease Notification (EDN) data, an internal CDC data set that compiles information from public health departments (e.g., follow-up exam outcomes) for immigrants and refugees with Class B1 designations (9) to determine the numbers and types of diagnostic tests received by patients (9). Among Indian immigrants, about 11% received a simple followup with no chest radiograph (CXR) or smears/cultures; 28% received a CXR only; 39% received both a CXR and smears/cultures; and 21.4% did not follow up. Among Mexican immigrants, about 8.5% received a simple followup; 39% received only a chest radiograph; 31% received both chest radiograph and smears/cultures; and 21.4% did not follow up. We applied these country-specific percentages to our cohorts to estimate the cost of followup.

*Hospitalization Costs*

Hospitalization costs were estimated based on the 2006–2011 National Inpatient Sample (23). The average total costs for a hospitalization for pulmonary TB were used. Costs were updated to 2013 dollars using the BLS medical consumer price index (24). The average length of stay for pulmonary TB is about 14 days, with an estimated cost of $22,000. For persons who are hospitalized, we estimated opportunity costs based on the average length of stay for pulmonary TB (14 days) in addition to the 12.5 days required for outpatient treatment.

*Employer Costs*

For H-1B employers, there are substantial costs associated with visa applications. Specifically, H-1B employers are responsible for paying a fraud detection fee ($500) and the American Competitiveness and Workforce Improvement Act of 1998 fee ($1,500) (10). The applicant filing fee ($325) does not need to be paid by the employer and is a cost that is assigned to the worker (10). Employers would incur additional visa application costs if a worker is diagnosed with active TB in India and elects to obtain treatment at a local healthcare site instead of the panel physician site, because such workers would have to wait for at least one year, reapply for a visa, and undergo a second medical exam by a panel physician after one year.

Employers who recruit H-2A and H-2B workers do not need to pay a per-applicant fee (10). Instead, these employers file the same petition for multiple applicants. Hence, we do not assess marginal forfeited visa fees for the employers when workers are diagnosed with TB and elect to obtain treatment at local sites.

# A5. India and Mexico Screening Processes and Costs

*Treatment Costs*

Of the eight panel physician sites in India, five provided information on TB-related exam fees. Costs for each item were averaged over all sites and converted to 2013 dollars. In Mexico, all panel physician sites provided information on TB-related exam fees.

In Mexico, panel physician exam fees are all-inclusive, consisting of screening, diagnosis, and treatment for TB as well as screening for other conditions as required by CDC’s Technical Instructions. We applied the percentage of TB-related costs in India to determine the portion of the panel physician exam fee that is attributable to TB-related activities in Mexico (50%).

Time Values

Opportunity costs were estimated for workers’ (and family members’) time spent on TB screening and treatment. We assumed that all persons would lose 1 day of wages to travel to the panel physician site for screening. Persons with clinical findings suggestive of TB would spend an additional 3 days for sputum smears and culture testing.

To compute the opportunity costs for persons with active TB who opt for treatment at the panel physician site, we assumed that 30% of 6 months of treatment would be spent on treatment-related activities and/or disease impairment. NIVs may live far from the panel physician site and require greater time to access services. For persons electing treatment at a local healthcare facility, we assumed they would spend the same number of hours obtaining treatment as if they were treated in the United States because they’d likely choose a treatment site closer to home.

Opportunity costs for Indian H-1B visa applicants were estimated using average wages reported for information technology managers with experience at varying levels. Annual salaries were averaged for workers at beginning, middle, and senior career levels (25). Opportunity costs associated with Indian family members (H-4 visa applicants) were computed using country-specific GDP adjusted for PPP (13).

For Mexican agricultural worker NIVs (H-2B), overseas wages for agricultural employees were used to estimate opportunity costs. To compute the opportunity costs for H-2B NIVs, we took the average of the following occupations: agricultural, hunting and forestry, hotel and restaurants, and other community, social, and personal services.

All wages are converted to 2013 dollars adjusted for PPP.

# A6. Additional Results and Sensitivity Analyses

We conducted the following sensitivity analyses to examine uncertainty in our key parameters:

1. Increased and decreased the proportion of NIVs with abnormal CXR or other signs and symptoms of TB at panel physician exams by varying the percentage of the immigrant rate used for India
2. Increased and decreased the proportion of NIVs with abnormal CXR or other signs and symptoms of TB at panel physician exams (50 to 150%) for Mexico
3. Increased and decreased the proportion of persons with Class B1 status who developed active TB (incident cases) by 50%
4. Varied the amount of opportunity (time) costs of screening and treatment at panel physician sites from 33% (lower end) and 167% (upper end) of baseline estimates
5. Calculated incremental cost-effectiveness ratios after excluding opportunity costs

The results of these sensitivity analyses are presented in the manuscript.

*NIVs with Abnormal CXRs or Other Signs and Symptoms of TB at Panel Physician Exams*

Because of the high degree of uncertainty in the total number of active TB cases in the United States among the Indian NIV cohort, we applied a correction factor to the fraction of immigrants with abnormal CXRs or other signs and symptoms of TB reported for immigrants. This correction factor would affect the number of NIVs (versus immigrants) that must provide sputum samples, the number of TB cases diagnosed (both in India and the United States) and the number arriving in the United States with Class B1 status (see Table A6.1). We also provided a sensitivity analysis for the Mexican NIV cohort (Table A6.2).

| **Table A6.1 Cost per Case Avoided by Correction Factor for Indian NIVs (versus Immigrants) with Abnormal CXRs or Other Signs and Symptoms of Tuberculosis** | | | |
| --- | --- | --- | --- |
| Percent of Immigrant  Abnormal CXR or Other Signs and Symptoms of Tuberculosis | Number of Total Prevalent and Incident TB Cases | Number of U.S. TB Cases Avoided | Cost per U.S. Case of TB Avoided |
| 20% | 45 | 28 | $743,690 |
| 30% | 67 | 41 | $496,897 |
| 40% | 90 | 55 | $373,501 |
| 50% | 112 | 69 | $299,463 |
| 60% | 134 | 82 | $252,358 |
| 70% | 157 | 97 | $213,804 |
| 80% | 179 | 110 | $188,952 |
| 90% | 202 | 125 | $166,643 |
| 100% | 224 | 138 | $151,388 |

Notes: The percentage of immigrants with abnormal CXRs or other signs and symptoms of tuberculosis in India was 3.4%. In this analysis, we applied a correction factor for Indian NIVs (workers and their adult family members) to adjust the Indian immigrant rate to be representative of NIVs (i.e. Indians arriving with H-1B or H-4 visas).

| **Table A6.2 Cost per Case Avoided by Correction Factor for Mexican NIVs (versus Immigrants) with Abnormal CXRs or Other Signs and Symptoms of Tuberculosis** | | | |
| --- | --- | --- | --- |
| Percent of Immigrant  Abnormal CXR or Other Signs and Symptoms of Tuberculosis | Number of Total Prevalent and Incident TB Cases | Number of U.S. TB Cases Avoided | Cost per U.S. Case of TB Avoided |
| 20% | 10 | 8 | $1,147,596 |
| 30% | 13 | 10 | $915,969 |
| 40% | 16 | 12 | $761,551 |
| 50% | 18 | 14 | $651,253 |
| 60% | 21 | 16 | $568,529 |
| 70% | 23 | 19 | $504,188 |
| 80% | 26 | 21 | $452,715 |
| 90% | 31 | 25 | $375,506 |
| 100% | 36 | 29 | $320,357 |
| 110% | 57 | 45 | $200,031 |
| 120% | 62 | 49 | $182,483 |
| 135% | 70 | 56 | $161,036 |
| 150% | 78 | 62 | $143,879 |

Notes: The percentage of immigrants with abnormal CXRs or other signs and symptoms of tuberculosis in Mexico was 2.2%. In this analysis, we applied a correction factor for Mexican NIVs (workers) to adjust the Mexican immigrant rate to be representative of NIVs. The upper bound was set greater than 100% in case applicants for temporary work visas would have higher TB prevalence than immigrant visa applicants.

We also computed additional costs for the following items: travel delay-related opportunity costs and disease transmission costs.

*Travel Delay-related Opportunity Costs*

Travel delay-related opportunity costs were calculated for persons who were diagnosed with active TB and elected to obtain treatment at panel physician sites. Travel delay-related opportunity costs were computed by taking the difference in wages between the home country and the United States. This figure was multiplied by 6 months of treatment (n=1,040 working hours). These costs are presented below but are not included in the main analysis (Table A6.3).

**Table A6.3 Overseas Opportunity (Time) Cost Values for Individuals with NIV Work Visas whose U.S. Entry is Delayed, by Country and Visa**

| **Component** | **India** | **Mexico (H-2A)** | **Mexico (H-2B)** |
| --- | --- | --- | --- |
| # of people delayed 6 months by obtaining treatment at panel physician site | 51.5 | 19.4 | 12.4 |
| Average hourly compensation in India during treatment | $37.43 | $2.46 | $3.27 |
| Average U.S. hourly compensation during treatment in USD | $56.25 | $13.34 | $13.52 |
| Difference in wages per hour | $18.82 | $10.89 | $10.25 |
| Total time cost of travel delay for those obtaining panel physician treatment | $1,008,168 | $219,097 | $78,800 |

Notes: Total time cost of travel delay for patients obtaining treatment at panel physician site was calculated by multiplying number of persons obtaining treatment at panel physician site x 1,041 working hours in a 6-month period x difference in wages per hour.

*Costs Associated with a Hypothetical Disease Transmission Scenario*

We also computed disease transmission costs over 25 years using a hypothetical disease transmission scenario (Table A6.4).

| **Table A6.4 Tuberculosis Disease Transmission Scenario in The United Cases, Additional U.S. Cases and Costs** | | | | |
| --- | --- | --- | --- | --- |
|  | India | | Mexico | |
|  | No Screening | Screening | No Screening | Screening |
| # of additional active U.S. TB cases due to disease transmission in the United States | 52 | 20 | 12 | 2 |
| Additional costs | $963,437 | $370,188 | $186,788 | $37,938 |

Notes: We assumed that there were 0.23 secondary cases in the United States per index case, with 1/3 occurring immediately, 1/3 after 5 years, and 1/3 after 25 years, and discounted the associated treatment costs at an annual rate of 3% (26). Since TB transmission from NIVs may occur both in NIVs’ home countries and after arrival in the United States, we assumed that half the transmission would occur in NIV home countries and half in the United States. Thus, the number of new cases per index case used in this analysis is 50% of the rates used in the cited references, since we are primarily interested in U.S.-specific transmission. This model assumed that the contact investigation would not be sufficient to completely prevent secondary cases.

# A7. Comparison to Screening Outcomes in Australia and the United Kingdom

Australia and the United Kingdom (UK) currently conduct screening programs for long term NIVs from India and report screening outcomes in annual reports (27, 28). In Table A7.1, we provided a comparison of screening outcomes for U.S.-bound immigrants from India to rates reported for all Indians (i.e. immigrants and long-term visa holders) relocating to the Australia and the UK. Australia and the UK also report the prevalence of TB diagnosed in their nonimmigrant worker population, although rates in long-term workers from India are not reported separately. The TB case detection rates reported for migrants to the UK are considerably greater than for migrants to Australia (5x greater for all Indians and 3.6x greater for nonimmigrant workers). The rates observed for U.S.-bound immigrants from India generally fall in between the UK and Australia case detection rates observed for all migrants from India.

**Table A7.1 Tuberculosis Case Detection Rates for U.S.-bound immigrants, UK-bound migrants, and Australia-bound migrants**

|  | Overseas (pre-departure) Cases Detected per 100,000 | Post-arrival Cases Detected per 100,000 |
| --- | --- | --- |
| U.S.-bound immigrants from India | 134 | 75 |
| Australia-bound immigrants and long term visitors from India (27) | 53 | 51^1^ |
| UK-bound immigrants and long term visitors from India (28) | ~270 | NA |
| Skilled workers in Australia (27) | 44 | NA |
| Workers in UK (28) | 160.5 | NA |

Notes: U.S.- United States, UK- United Kingdom

^1^This value is the case detection rate in all new arrivals from India in their first year in Australia based on data from the national surveillance system. In comparison, the data for U.S.-bound immigrants is limited to immigrants with abnormal chest radiographs or other signs and symptoms of tuberculosis at their follow-up evaluation after U.S. arrival.

**References**

1. U.S. Congress. U.S. Code of Laws. Title 8 and Title 42. 42 USC 252: <https://www.gpo.gov/fdsys/pkg/USCODE-2011-title42/html/USCODE-2011-title42-chap6A-subchapII-partC-sec252.htm>; 8 USC 1182: <https://www.gpo.gov/fdsys/pkg/USCODE-2012-title8/html/USCODE-2012-title8-chap12-subchapII-partII-sec1182.htm>; 8 USC 1222: <https://www.gpo.gov/fdsys/pkg/USCODE-2012-title8/html/USCODE-2012-title8-chap12-subchapII-partIV-sec1222.htm>. [Accessed March 5, 2018].

2. U.S. Centers for Disease Control and Prevention. Division of Global Migration and Quarantine. Available from: <http://www.cdc.gov/ncezid/dgmq>. [Accessed March 4, 2015].

3. U.S. Centers for Disease Control and Prevention. Tuberculosis Screening and Treatment Technical Instructions (TB TIs) using Cultures and Directly Observed Therapy (DOT) for Panel Physicians. Atlanta, GA: Centers for Disease Control and Prevention, U.S. Dept of Health and Human Services; 2013. <https://www.cdc.gov/immigrantrefugeehealth/exams/ti/panel/tuberculosis-panel-technical-instructions.html> [Accessed March 5, 2018].

4. World Health Organization. Tuberculosis country profiles. Available from: <http://www.who.int/tb/country/data/profiles/en/>. [Accessed March 2014].

5. Office of Immigration Statistics. 2012 Handbook of Immigration Statistics. Available from: <http://www.dhs.gov/sites/default/files/publications/ois_yb_2012.pdf>. [Accessed September 2014].

6. U.S. Department of State. Nonimmigrant Visa Statistics. Available from: <http://travel.state.gov/content/visas/en/law-and-policy/statistics/non-immigrant-visas.html>. [Accessed March 2015].

7. U.S. Centers for Disease Control and Prevention. Reported Tuberculosis in the United States, 2012. Atlanta, GA: U.S. Department of Health and Human Services, CDC, October 2013.

8. U.S. Centers for Disease Control and Prevention. Reported Tuberculosis in the United States, 2014. Atlanta, GA: U.S. Department of Health and Human Services, CDC, October 2015.

9. Lee D, Philen R, Wang Z, McSpadden P, Posey DL, Ortega LS, et al. Disease Surveillance Among Newly Arriving Refugees and Immigrants — Electronic Disease Notification System, United States, 2009. Morbidity and Mortality Weekly Report Surveillance Summaries. 2013;62(SS07):1-20.

10. U.S. Citizenship and Immigration Services. Characteristics of H-1B Specialty Occupation Workers. U.S. Department of Homeland Security; 2013. <https://www.uscis.gov/sites/default/files/USCIS/Resources/Resources%20for%20Congress/H-1B_Characteristics_Report_FY_2013_826_KB.pdf>. [Accessed March 1, 2018].

11. U.S. Bureau of Labor Statistics. Consumer Price Index. <https://www.bls.gov/cpi/tables/supplemental-files/home.htm> [Accessed March 5, 2018].

12. U.S. Bureau of Labor Statistics. May 2012 National Occupational Employment and Wage Estimates United States. <https://www.bls.gov/oes/tables.htm>. [Accessed Mrch 5, 2018].

13. The World Bank. GDP per capita, PPP (current international $) <https://data.worldbank.org/indicator/NY.GDP.PCAP.PP.CD> [Accessed March 2014].

14. Posey D. Centers for Disease Control and Prevention [personal communication] 2015.

15. Wingate LT, Coleman MS, Posey DL, Zhou W, Olson CK, Maskery B, et al. Cost-effectiveness of Screening and Treating Foreign-born Students for Tuberculosis before Entering the United States. Plos One. 2015;10(4):e0124116.

16. Centers for Medicare and Medicaid Services. 2013 Clinical Laboratory Fee Schedule, <https://www.cms.gov/Medicare/Medicare-Fee-for-Service-Payment/ClinicalLabFeeSched/Clinical-Laboratory-Fee-Schedule-Files.html>. Accessed 2/20/2018.; 2013.

17. Chaulk CP, Kazandjian VA. Directly observed therapy for treatment completion of pulmonary tuberculosis: Consensus Statement of the Public Health Tuberculosis Guidelines Panel. JAMA : the Journal of the American Medical Association. 1998;279(12):943-8.

18. Escobedo M. Centers for Disease Control and Prevention. [personal communication] 2015. .

19. U.S. Centers for Disease Control and Prevention. CDC WONDER Database Online Tuberculosis Information System <https://wonder.cdc.gov/TB-v2016.html> [Accessed March 5, 2018].

20. Santos A, McGuckin N, Nakamoto HY, Gray D, Liss S. Summary of Travel Trends: 2009 National Household Travel Survey. Washington, D.C.: Federal Highway Administration, U.S. Department of Transportation; 2009. <http://nhts.ornl.gov/2009/pub/stt.pdf> [Accessed March 1, 2018].

21. Smith ML, Dickerson JB, Wendel ML, Ahn S, Pulczinski JC, Drake KN, et al. The utility of rural and underserved designations in geospatial assessments of distance traveled to healthcare services: Implications for public health research and practice. J Environ Public Health. 2013;2013:960157.

22. Liu Y, Weinberg MS, Ortega LS, Painter JA, Maloney SA. Overseas Screening for Tuberculosis in U.S.-Bound Immigrants and Refugees. The New England Journal of Medicine. 2009;360:2406-15.

23. HCUP Nationwide Inpatient Sample (NIS). Healthcare Cost and Utilization Project (HCUP). 2006-2010. Agency for Healthcare Research and Quality, Rockville, MD. [www.hcup-us.ahrq.gov/nisoverview.jsp](file:///\\cdc.gov\private\M327\wqm7\Bisma%20projects\TB%20long%20term%20workers\www.hcup-us.ahrq.gov\nisoverview.jsp) [Accessed March 2013].

24. U.S. Bureau of Labor Statistics. Medical Consumer Price Index. Available from: <http://www.bls.gov/cpi/>. [Accessed May 2014].

25. Kelly Services India. 2013 Salary Guide. Available from: <http://www.kellyservices.co.in/uploadedFiles/India_-_Kelly_Services/Documents/India%20salary%202013%2014.pdf>. [Accessed May 2015].

26. Shepardson D, Marks SM, Chesson H, Kerrigan A, Holland DP, Scott N, et al. Cost-effectiveness of a 12-dose regimen for treating latent tuberculous infection in the United States. International Journal of Tuberculosis and Lung Disease. 2013;17(12):1531-7.

27. Australia Immigration Health Policy and Performance Branch Health Services and Policy Division. 2016 Tuberculosis identified through offshore pre-migration health screening 2015.

28. Public Health England, TB Screening Unit. (September 2016) UK pre-entry tuberculosis screening report 2015. PHE publications gateway number: 2016324. <https://www.gov.uk/government/uploads/system/uploads/attachment_data/file/555150/UK_pre-entry_tuberculosis_screening_2015_GTW230916.pdf> [Accessed March 1, 2018].

^
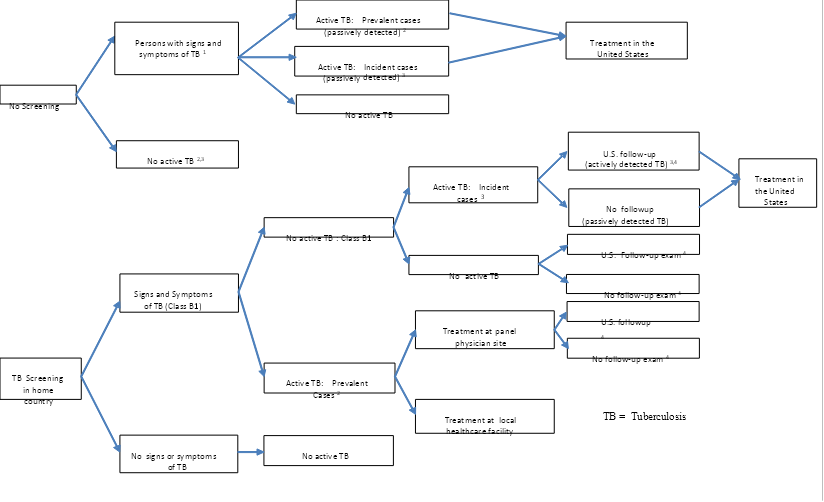
^

^
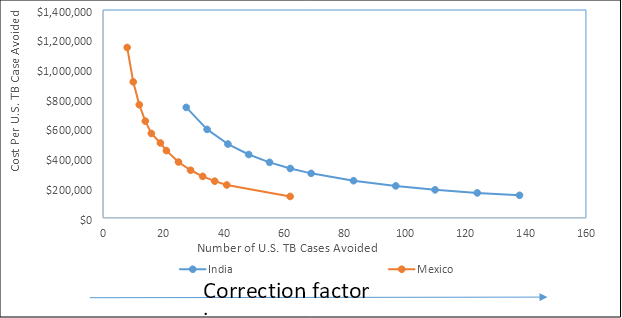
^
